# Supplementary material for: Variomes: a high recall search engine to support the curation of genomic variants
Source: Bioinformatics. 2022 Mar 11;38(9):2595–601. doi: 10.1093/bioinformatics/btac146 (PMC9048643; doi:10.1093/bioinformatics/btac146)
Supplement: btac146_Supplementary_Data [file btac146_supplementary_data.pdf]

Queries for the comparison between LitVar and Variomes

Gene	Variant
BRCA1	R7C
BRCA1	V11A
BRCA1	I15L
BRCA1	I15T
BRCA1	M18K
BRCA1	M18T
BRCA1	I21V
BRCA1	L22S
BRCA1	C24R
BRCA1	L28P
BRCA1	I31M
BRCA1	T37K
BRCA1	T37R
BRCA1	K38N
BRCA1	C39S
BRCA1	C39R
BRCA1	C39G
BRCA1	C39A
BRCA1	C39Y
BRCA1	H41A
BRCA1	H41R
BRCA1	I42V
BRCA1	C44F
BRCA1	K45Q
BRCA1	K45T
BRCA1	K45N
BRCA1	C47G
BRCA1	L51A
BRCA1	L52F
BRCA1	K56R
BRCA1	C61G
BRCA1	C61A
BRCA1	L63F
BRCA1	C64R
BRCA1	C64G
BRCA1	C64A
BRCA1	C64Y
BRCA1	D67Y
BRCA1	D67E
BRCA1	I68K
BRCA1	R71G
BRCA1	S72R
BRCA1	T77M
BRCA1	L87V
BRCA1	I89T

BRCA1	I90T
BRCA1	D96N
BRCA1	G98R
BRCA1	Y105C
BRCA1	I124V
BRCA1	S127N
BRCA1	N132K
BRCA1	P142H
BRCA1	E143K
BRCA1	E143*
BRCA1	Q155E
BRCA1	T164P
BRCA1	R170Q
BRCA1	Y179C
BRCA1	S186Y
BRCA1	V191I
BRCA1	L204F
BRCA1	R213G
BRCA1	A221S
BRCA1	D232N
BRCA1	H239R
BRCA1	L246V
BRCA1	R252H
BRCA1	V271M
BRCA1	G275S
BRCA1	T276R
BRCA1	A280G
BRCA1	Q284R
BRCA1	M297L
BRCA1	M297I
BRCA1	E300D
BRCA1	R315G
BRCA1	S316G
BRCA1	A322P
BRCA1	R331S
BRCA1	R332Q
BRCA1	P334H
BRCA1	P334L
BRCA1	W353*
BRCA1	K355R
BRCA1	Q356R
BRCA1	D369N
BRCA1	W372*
BRCA1	T374I
BRCA1	I379M
BRCA1	K408E
BRCA1	D411N
BRCA1	D411E

BRCA1	N417S
BRCA1	D420Y
BRCA1	E421K
BRCA1	E445Q
BRCA1	E445*
BRCA1	S454N
BRCA1	I456T
BRCA1	F461L
BRCA1	G462R
BRCA1	R466G
BRCA1	N473S
BRCA1	H476R
BRCA1	F486L
BRCA1	R496C
BRCA1	R496H
BRCA1	K503R
BRCA1	R504C
BRCA1	R504H
BRCA1	R507I
BRCA1	D522N
BRCA1	K527N
BRCA1	T539M
BRCA1	N550H
BRCA1	Q563*
BRCA1	P568L
BRCA1	I571T
BRCA1	E575K
BRCA1	E597K
BRCA1	H601R
BRCA1	N609S
BRCA1	R612G
BRCA1	A622V
BRCA1	E624K
BRCA1	V627I
BRCA1	S628R
BRCA1	S632N
BRCA1	D642H
BRCA1	S645Y
BRCA1	K654Q
BRCA1	M658I
BRCA1	H662R
BRCA1	L668F
BRCA1	Q687P
BRCA1	D693N
BRCA1	D695N
BRCA1	D695Y
BRCA1	S708Y
BRCA1	N723D

BRCA1	P727L
BRCA1	R728K
BRCA1	E736A
BRCA1	S741C
BRCA1	S741F
BRCA1	E765G
BRCA1	V772A
BRCA1	S784L
BRCA1	V788I
BRCA1	T796I
BRCA1	N799K
BRCA1	Q804H
BRCA1	A807S
BRCA1	N810Y
BRCA1	H816R
BRCA1	K820E
BRCA1	D825Y
BRCA1	T826K
BRCA1	R841W
BRCA1	E842G
BRCA1	Y856H
BRCA1	F861C
BRCA1	K862E
BRCA1	R866C
BRCA1	P871L
BRCA1	G890R
BRCA1	G890V
BRCA1	N909I
BRCA1	V920A
BRCA1	I925L
BRCA1	D940Y
BRCA1	T967S
BRCA1	R979C
BRCA1	M1008V
BRCA1	M1008I
BRCA1	M1014V
BRCA1	N1016D
BRCA1	I1019V
BRCA1	S1027N
BRCA1	R1028C
BRCA1	R1028H
BRCA1	E1038G
BRCA1	S1040N
BRCA1	I1044V
BRCA1	E1060*
BRCA1	E1060A
BRCA1	R1074G
BRCA1	R1076T

BRCA1	L1086S
BRCA1	V1088D
BRCA1	P1099L
BRCA1	S1101N
BRCA1	C1103F
BRCA1	H1105N
BRCA1	V1116L
BRCA1	E1134*
BRCA1	P1136R
BRCA1	M1137T
BRCA1	S1140G
BRCA1	Q1144*
BRCA1	Q1144H
BRCA1	P1150S
BRCA1	D1152N
BRCA1	D1155H
BRCA1	E1172G
BRCA1	V1176D
BRCA1	V1181I
BRCA1	K1183R
BRCA1	T1196K
BRCA1	Q1200H
BRCA1	G1201S
BRCA1	R1203Q
BRCA1	G1205R
BRCA1	E1214K
BRCA1	S1218C
BRCA1	E1219D
BRCA1	E1222*
BRCA1	N1236K
BRCA1	P1238R
BRCA1	P1238L
BRCA1	V1247I
BRCA1	T1249S
BRCA1	E1250K
BRCA1	S1266T
BRCA1	I1275V
BRCA1	H1284R
BRCA1	A1293V
BRCA1	T1310K
BRCA1	E1346K
BRCA1	R1347G
BRCA1	T1349P
BRCA1	T1349R
BRCA1	T1349M
BRCA1	M1361L
BRCA1	C1372Y
BRCA1	V1378I

BRCA1	Q1395*
BRCA1	Q1396*
BRCA1	M1400V
BRCA1	H1402Y
BRCA1	L1404R
BRCA1	I1405V
BRCA1	L1407P
BRCA1	E1419Q
BRCA1	H1421Y
BRCA1	R1443G
BRCA1	R1443*
BRCA1	N1468H
BRCA1	T1485I
BRCA1	K1487R
BRCA1	R1495M
BRCA1	S1497A
BRCA1	W1508*
BRCA1	S1512I
BRCA1	Y1522C
BRCA1	I1529V
BRCA1	V1534M
BRCA1	D1546N
BRCA1	D1546Y
BRCA1	T1561I
BRCA1	L1564P
BRCA1	E1586D
BRCA1	R1589H
BRCA1	S1613G
BRCA1	S1613C
BRCA1	P1614S
BRCA1	A1623G
BRCA1	M1628V
BRCA1	M1628T
BRCA1	P1637L
BRCA1	E1644G
BRCA1	N1647K
BRCA1	S1651F
BRCA1	M1652T
BRCA1	M1652I
BRCA1	V1653M
BRCA1	S1655A
BRCA1	S1655F
BRCA1	F1662S
BRCA1	M1663L
BRCA1	M1663K
BRCA1	L1664P
BRCA1	V1665M
BRCA1	A1669S

BRCA1	E1682K
BRCA1	E1682V
BRCA1	T1685A
BRCA1	T1685I
BRCA1	M1689T
BRCA1	M1689R
BRCA1	T1691K
BRCA1	T1691I
BRCA1	D1692N
BRCA1	D1692H
BRCA1	D1692Y
BRCA1	E1694K
BRCA1	E1694*
BRCA1	F1695L
BRCA1	V1696L
BRCA1	C1697R
BRCA1	E1698R
BRCA1	R1699W
BRCA1	R1699Q
BRCA1	R1699L
BRCA1	T1700A
BRCA1	G1706E
BRCA1	G1706A
BRCA1	A1708E
BRCA1	A1708V
BRCA1	V1713A
BRCA1	V1714G
BRCA1	S1715R
BRCA1	S1715C
BRCA1	S1715N
BRCA1	S1715R
BRCA1	W1718S
BRCA1	W1718C
BRCA1	T1720A
BRCA1	S1722F
BRCA1	R1726G
BRCA1	K1727*
BRCA1	N1730S
BRCA1	H1732D
BRCA1	D1733G
BRCA1	F1734S
BRCA1	V1736A
BRCA1	V1736G
BRCA1	R1737*
BRCA1	G1738R
BRCA1	G1738E
BRCA1	D1739Y
BRCA1	D1739G

BRCA1	D1739V
BRCA1	D1739E
BRCA1	V1741G
BRCA1	N1745R
BRCA1	H1746N
BRCA1	H1746D
BRCA1	P1749R
BRCA1	R1751*
BRCA1	R1751Q
BRCA1	R1751P
BRCA1	A1752P
BRCA1	A1752V
BRCA1	R1753T
BRCA1	F1761S
BRCA1	G1763V
BRCA1	L1764P
BRCA1	I1766S
BRCA1	P1771R
BRCA1	P1771L
BRCA1	T1773S
BRCA1	T1773I
BRCA1	N1774R
BRCA1	M1775K
BRCA1	M1775R
BRCA1	P1776H
BRCA1	D1778N
BRCA1	D1778Y
BRCA1	D1778G
BRCA1	L1780P
BRCA1	M1783L
BRCA1	M1783T
BRCA1	Q1785E
BRCA1	C1787S
BRCA1	G1788D
BRCA1	G1788V
BRCA1	A1789T
BRCA1	A1789S
BRCA1	E1794D
BRCA1	G1803A
BRCA1	V1804D
BRCA1	H1805P
BRCA1	P1806A
BRCA1	V1808A
BRCA1	V1809F
BRCA1	V1809A
BRCA1	V1810G
BRCA1	Q1811R
BRCA1	P1812A

BRCA1	D1818G
BRCA1	N1819S
BRCA1	A1823T
BRCA1	A1830T
BRCA1	V1833M
BRCA1	R1835P
BRCA1	E1836K
BRCA1	E1836*
BRCA1	W1837R
BRCA1	W1837G
BRCA1	W1837C
BRCA1	V1838E
BRCA1	S1841R
BRCA1	S1841N
BRCA1	A1843P
BRCA1	L1844R
BRCA1	D1851E
BRCA1	Y1853C
BRCA1	L1854P
BRCA1	P1856S
BRCA1	I1858L
BRCA1	P1859R
BRCA2	R18H
BRCA2	G25R
BRCA2	I27V
BRCA2	W31R
BRCA2	W31C
BRCA2	Y42C
BRCA2	E49*
BRCA2	N56T
BRCA2	K63N
BRCA2	A75P
BRCA2	N108H
BRCA2	Q147*
BRCA2	H150R
BRCA2	P168T
BRCA2	P168A
BRCA2	G173C
BRCA2	G173V
BRCA2	R174C
BRCA2	D191G
BRCA2	D191V
BRCA2	W194*
BRCA2	S196N
BRCA2	S206C
BRCA2	T207A
BRCA2	L209F
BRCA2	V211I

BRCA2	T149A
BRCA2	T251R
BRCA2	S286P
BRCA2	N289H
BRCA2	N319T
BRCA2	S326R
BRCA2	K327E
BRCA2	N372H
BRCA2	P375S
BRCA2	S384F
BRCA2	W395G
BRCA2	E462G
BRCA2	I505T
BRCA2	K513R
BRCA2	N517S
BRCA2	C554W
BRCA2	T582P
BRCA2	N588D
BRCA2	N588S
BRCA2	T598A
BRCA2	G602R
BRCA2	K607T
BRCA2	T630I
BRCA2	P655R
BRCA2	D707Y
BRCA2	Q713L
BRCA2	M784V
BRCA2	Y792*
BRCA2	D806H
BRCA2	N886I
BRCA2	V894I
BRCA2	M927V
BRCA2	L929S
BRCA2	D935N
BRCA2	N987I
BRCA2	N991D
BRCA2	T1011R
BRCA2	L1019V
BRCA2	K1057R
BRCA2	N1102Y
BRCA2	A1170V
BRCA2	S1172L
BRCA2	R1190W
BRCA2	G1194D
BRCA2	N1228D
BRCA2	C1265S
BRCA2	I1275M
BRCA2	N1279S

BRCA2	D1280V
BRCA2	K1286E
BRCA2	C1290Y
BRCA2	V1306I
BRCA2	E1308*
BRCA2	D1352Y
BRCA2	T1354M
BRCA2	C1365Y
BRCA2	Q1396R
BRCA2	T1414M
BRCA2	D1420N
BRCA2	D1420Y
BRCA2	S1424C
BRCA2	T1430A
BRCA2	K1434I
BRCA2	L1457F
BRCA2	D1513N
BRCA2	F1524V
BRCA2	G1529R
BRCA2	C1573Y
BRCA2	E1593D
BRCA2	V1643A
BRCA2	K1690N
BRCA2	S1733F
BRCA2	S1760A
BRCA2	G1771D
BRCA2	N1805S
BRCA2	P1819S
BRCA2	S1871N
BRCA2	N1878K
BRCA2	N1880K
BRCA2	S1882*
BRCA2	D1902N
BRCA2	L1904V
BRCA2	N1910I
BRCA2	C1913*
BRCA2	T1915M
BRCA2	H1918Y
BRCA2	I1929V
BRCA2	H1966Y
BRCA2	Q1994*
BRCA2	D2005V
BRCA2	S2006R
BRCA2	R2034C
BRCA2	G2044A
BRCA2	N2048I
BRCA2	H2074N
BRCA2	T2097M

BRCA2	L2106P
BRCA2	R2108C
BRCA2	R2108H
BRCA2	N2113S
BRCA2	V2138F
BRCA2	Y2222C
BRCA2	T2250A
BRCA2	G2274V
BRCA2	I2285V
BRCA2	F2293L
BRCA2	D2312V
BRCA2	R2318Q
BRCA2	R2336H
BRCA2	A2351G
BRCA2	G2353R
BRCA2	Q2384K
BRCA2	L2396F
BRCA2	F2406L
BRCA2	K2411T
BRCA2	R2418G
BRCA2	N2436I
BRCA2	K2446E
BRCA2	A2466V
BRCA2	K2472T
BRCA2	L2480V
BRCA2	S2483G
BRCA2	S2483N
BRCA2	R2488K
BRCA2	R2488S
BRCA2	D2489G
BRCA2	I2490T
BRCA2	Q2491*
BRCA2	R2494Q
BRCA2	K2496T
BRCA2	R2500T
BRCA2	R2502C
BRCA2	R2502H
BRCA2	R2502P
BRCA2	F2504L
BRCA2	G2508S
BRCA2	L2510P
BRCA2	L2512F
BRCA2	T2515I
BRCA2	R2520Q
BRCA2	I2521V
BRCA2	I2521T
BRCA2	S2522F
BRCA2	G2528E

BRCA2	S2533C
BRCA2	A2534V
BRCA2	H2537R
BRCA2	T2542R
BRCA2	V2545I
BRCA2	K2547E
BRCA2	F2562L
BRCA2	D2566Y
BRCA2	E2571G
BRCA2	L2581W
BRCA2	G2584D
BRCA2	G2585R
BRCA2	P2589H
BRCA2	N2591S
BRCA2	A2595S
BRCA2	R2602T
BRCA2	T2607P
BRCA2	G2609D
BRCA2	D2611G
BRCA2	H2623R
BRCA2	W2626C
BRCA2	I2627F
BRCA2	P2639A
BRCA2	A2643G
BRCA2	A2643V
BRCA2	N2644S
BRCA2	L2647P
BRCA2	L2653P
BRCA2	L2654P
BRCA2	Q2655R
BRCA2	R2659G
BRCA2	R2659K
BRCA2	R2659T
BRCA2	Y2660D
BRCA2	E2663K
BRCA2	E2663V
BRCA2	D2665G
BRCA2	R2666T
BRCA2	S2670L
BRCA2	I2672V
BRCA2	I2675V
BRCA2	M2676T
BRCA2	R2678G
BRCA2	D2679Y
BRCA2	D2679G
BRCA2	D2680G
BRCA2	T2681R
BRCA2	K2684R

BRCA2	L2686P
BRCA2	V2687F
BRCA2	L2688P
BRCA2	C2689*
BRCA2	S2691F
BRCA2	S2695L
BRCA2	S2697N
BRCA2	A2698T
BRCA2	I2700L
BRCA2	S2704F
BRCA2	N2706S
BRCA2	D2712N
BRCA2	D2712V
BRCA2	Q2714R
BRCA2	A2717S
BRCA2	I2718T
BRCA2	L2721H
BRCA2	L2721R
BRCA2	T2722R
BRCA2	D2723H
BRCA2	D2723G
BRCA2	D2723A
BRCA2	G2724V
BRCA2	Y2726C
BRCA2	V2728I
BRCA2	V2728L
BRCA2	K2729N
BRCA2	A2730P
BRCA2	A2730V
BRCA2	V2739I
BRCA2	G2748D
BRCA2	I2752F
BRCA2	A2770T
BRCA2	M2775T
BRCA2	M2775R
BRCA2	N1730Y
BRCA2	R2784W
BRCA2	R2784Q
BRCA2	A2786P
BRCA2	R2787C
BRCA2	R2787H
BRCA2	W2788R
BRCA2	W2788S
BRCA2	T2790S
BRCA2	L2792P
BRCA2	G2793R
BRCA2	G2793E
BRCA2	F2794L

BRCA2	P2800S
BRCA2	P2800R
BRCA2	S2807L
BRCA2	S2810G
BRCA2	D2811G
BRCA2	G2812E
BRCA2	G2813E
BRCA2	V2815I
BRCA2	V2818I
BRCA2	V2820L
BRCA2	I2821T
BRCA2	I2828V
BRCA2	Q2829L
BRCA2	S2835P
BRCA2	I2840V
BRCA2	R2842C
BRCA2	R2842H
BRCA2	R2842L
BRCA2	N2843S
BRCA2	E2847K
BRCA2	K2849E
BRCA2	E2850K
BRCA2	E2856A
BRCA2	Q2858K
BRCA2	Q2858R
BRCA2	A2864V
BRCA2	L2865V
BRCA2	L2865*
BRCA2	T2867P
BRCA2	F2873C
BRCA2	P2883S
BRCA2	R2888C
BRCA2	R2888P
BRCA2	R2888L
BRCA2	A2889S
BRCA2	L2890I
BRCA2	R2896H
BRCA2	G2901D
BRCA2	V2908G
BRCA2	A2911V
BRCA2	S2988N
BRCA2	G2919V
BRCA2	S2922G
BRCA2	Q2925R
BRCA2	Q2925H
BRCA2	L2936M
BRCA2	L2936F
BRCA2	A2942T

BRCA2	I2944F
BRCA2	E2947K
BRCA2	K2950N
BRCA2	A2951T
BRCA2	D2965H
BRCA2	V2969M
BRCA2	L2972W
BRCA2	R2973C
BRCA2	R2973H
BRCA2	K2982Q
BRCA2	S2988G
BRCA2	W2990*
BRCA2	R2991H
BRCA2	E3002K
BRCA2	E3002D
BRCA2	Y3006*
BRCA2	L3011P
BRCA2	T3013I
BRCA2	S3020C
BRCA2	Q3026E
BRCA2	A3028P
BRCA2	A3029T
BRCA2	A3029V
BRCA2	Q3034R
BRCA2	Y3035S
BRCA2	Y3035C
BRCA2	P3039L
BRCA2	V3040I
BRCA2	R3052W
BRCA2	R3052Q
BRCA2	P3054H
BRCA2	S3058G
BRCA2	K3059E
BRCA2	P3063S
BRCA2	D3064N
BRCA2	D3064Y
BRCA2	F3065L
BRCA2	Q3066E
BRCA2	E3071D
BRCA2	V3072E
BRCA2	D3073G
BRCA2	G3076E
BRCA2	G3076V
BRCA2	V3079I
BRCA2	V3081A
BRCA2	K3083E
BRCA2	K3083N
BRCA2	A3088V

BRCA2	V3091I
BRCA2	Y3092S
BRCA2	Y3092C
BRCA2	D3095E
BRCA2	E3096*
BRCA2	Y3098H
BRCA2	L3101V
BRCA2	L3101R
BRCA2	I3103V
BRCA2	I3103M
BRCA2	H3117P
BRCA2	M3118T
BRCA2	S3123G
BRCA2	N3124I
BRCA2	L3125H
BRCA2	Q3126*
BRCA2	W3127*
BRCA2	P3129A
BRCA2	G3134V
BRCA2	D3142G
BRCA2	P3150L
BRCA2	E3152K
BRCA2	E3152G
BRCA2	G3153A
BRCA2	F3159L
BRCA2	E3167A
BRCA2	D3170G
BRCA2	M3181R
BRCA2	D3188N
BRCA2	C3198R
BRCA2	T3211K
BRCA2	K3257R
BRCA2	R3276S
BRCA2	Y3308*
BRCA2	E3309*
BRCA2	K3326*
BRCA2	T3349A
BRCA2	I3412V

Manual evaluation of the results retrieved by Variomes and LitVar for 10 random queries

variant	PMID/PMCID	retrieved by Variomes	retrieved by LitVar	relevant	comment
BRCA1 (L246V)	22753008	TRUE	FALSE	TRUE	Leu246Val in Discussion table; functional assay (yeast and mouse cell lines), pathogenicity assessment
BRCA1 (L246V)	26183948	TRUE	TRUE	TRUE	L246V in Results table; germline mutation testing, spectrum
BRCA1 (L246V)	32055015	TRUE	FALSE	TRUE	c.736T>G in Discussion; genetic testing report format evaluation
BRCA1 (L246V)	19200354	TRUE	TRUE	TRUE	p.L246V in Results; germline mutation testing, pathogenicity assessment
BRCA1 (L246V)	21990134	TRUE	TRUE	TRUE	L246V in Main body table (Review type publication); pathogenicity assessment, classification model
BRCA1 (L246V)	24212087	TRUE	TRUE	TRUE	c.736T>G in Introduction; functional assay evaluation
BRCA1 (L246V)	28398198	TRUE	TRUE	TRUE	L246V in Results table; functional assay (human cell line), therapy response, pathogenicity assessment
BRCA1 (L246V)	28651617	TRUE	TRUE	TRUE	p.Leu246Val in Results table; gene sequencing panel and workflow evaluation
BRCA1 (L246V)	26246475	TRUE	TRUE	TRUE	L246V in Results and Discussion; functional assay (human cell lines)
BRCA1 (L246V)	19818148	TRUE	TRUE	TRUE	L246V in Results table; germline mutation testing, spectrum
BRCA1 (I1044V)	22753008	TRUE	TRUE	TRUE	Ile1044Val in Discussion table; functional assay (yeast and mouse cell lines), pathogenicity assessment
BRCA1 (I1044V)	21990134	TRUE	FALSE	TRUE	I1044V in Main body table (Review type publication); pathogenicity assessment, classification model
BRCA2 (V3091I)	21671020	TRUE	TRUE	TRUE	V3091I in Abstract, Materials and Methods, Results, Discussion; functional assay (human cell lines), pathogenicity assessment
BRCA2 (V3091I)	24323938	TRUE	FALSE	TRUE	p.Val3091Ile in Main body table; functional assay (human and non-human cell lines), pathogenicity assessment
BRCA2 (V3091I)	32438681	TRUE	FALSE	TRUE	p.(Val3091Ile) in Results table; germline mutation testing, spectrum
BRCA2 (V3091I)	26681674	TRUE	TRUE	TRUE	V3091I in Results; tumor mutation testing
BRCA2 (G2813E)	23108138	TRUE	TRUE	TRUE	G2813E in Results table and Discussion; germline mutation testing, functional assay (hamster cell line), pathogenicity assessment
BRCA2 (G2813E)	32042831	TRUE	FALSE	TRUE	Gly2813Glu in Abstract and Results; pathogenicity assessment
BRCA2 (G2813E)	24323938	TRUE	FALSE	TRUE	p.Gly2813Glu in Main body table; functional assay (human and non-human cell lines) and pathogenicity assessment
BRCA1 (S1497A)	22753008	TRUE	FALSE	TRUE	p.Ser1497Ala in Materials and Methods; functional assay (yeast and mouse cell lines), pathogenicity assessment
BRCA1 (S1497A)	19770520	TRUE	FALSE	TRUE	S1497A in Abstract and Results; functional assay (mESC)
BRCA1 (S1497A)	19683496	TRUE	FALSE	TRUE	S1497A in Abstract, Results and Discussion; functional assay (mESC)
BRCA1 (S1497A)	28166811	TRUE	FALSE	TRUE	p.Ser1497Ala in Results; pathogenicity assessment, classification model
BRCA1 (S1497A)	21706030	TRUE	FALSE	TRUE	S1497A in Results; functional assay (human cell lines and mice)
BRCA2 (R3052W)	29884841	TRUE	TRUE	TRUE	R3052W in Results table; pathogenicity assessment, classification model
BRCA2 (R3052W)	32856869	TRUE	FALSE	TRUE	p.(Arg3052Trp) in Results table; germline and somatic tumor mutation testing, spectrum
BRCA2 (R3052W)	32438681	TRUE	FALSE	TRUE	p.(Arg3052Trp) in Results table; germline mutation testing, spectrum
BRCA2 (R3052W)	21520273	TRUE	TRUE	TRUE	R3052W in Abstracts, Results table and Discussion; germline mutation testing, pathogenicity assessment
BRCA2 (R3052W)	PMC6778823	TRUE	FALSE	TRUE	c.9154C>T in Main body (Poster Abstracts); functional assay (patient-derived cells)
BRCA2 (R3052W)	33233347	TRUE	FALSE	TRUE	p.(Arg3052Trp) in Results table; tumor mutation testing, spectrum, workflow evaluation
BRCA2 (R3052W)	31745186	TRUE	TRUE	TRUE	c.9154C > T in Results table; gene sequencing panel and workflow evaluation
BRCA2 (R3052W)	32322110	TRUE	FALSE	TRUE	R3052W in Appendix table; pathogenicity assessment, classification model and workflow
BRCA2 (R3052W)	21990134	TRUE	FALSE	TRUE	R3052W in Main body table (Review type publication); pathogenicity assessment, classification model
BRCA2 (R3052W)	23108138	TRUE	TRUE	TRUE	R3052W in Results table; germline mutation testing, functional assay (hamster cell line), pathogenicity assessment
BRCA2 (R3052W)	28283652	TRUE	TRUE	TRUE	R3052W in Results; germline mutation testing, functional assay (mESC, hamster and human cell lines), pathogenicity assessment
BRCA2 (R3052W)	24323938	TRUE	TRUE	TRUE	p.Arg3052Trp in Main body (Review); functional assay (human and non-human cell lines) and pathogenicity assessment
BRCA2 (R3052W)	25232094	TRUE	TRUE	TRUE	R3052W in Results and Discussion; human germinal and tumor variomes functional analysis

BRCA2 (R3052W)	29988080	TRUE	FALSE	TRUE	p.Arg3052Trp in Materials and Methods table; functional assay (mESC), pathogenicity assessment
BRCA2 (R3052W)	31360874	TRUE	TRUE	TRUE	c.9154C>T in Results; germline mutation testing, spectrum, functional assay (patient-derived cells),
BRCA2 (R3052W)	19200354	TRUE	TRUE	TRUE	p.R3052W in Abstract, Materials and Methods and Results; germline mutation testing, pathogenicity assessment
BRCA2 (R3052W)	30728895	TRUE	TRUE	TRUE	p.Arg3052Trp in Main body table; pathogenenicity assessment, reclassification
BRCA2 (R3052W)	18607349	TRUE	TRUE	TRUE	R3052W in Results and Discussion; functional assay (mESC)
BRCA2 (R3052W)	20104584	TRUE	TRUE	TRUE	R3052W in Results and Discussion; pathogenicity assessment, germline mutation testing
BRCA2 (R3052W)	32444794	TRUE	FALSE	TRUE	R3052W in Discussion; functional assay (human cell line) and pathogenicity assessment
BRCA2 (R3052W)	25146914	TRUE	TRUE	TRUE	p.Arg3052Trp in Results and Discussion; functional assay (E. Coli and mESC)
BRCA2 (R3052W)	33563323	TRUE	FALSE	TRUE	R3052W in Methods and Materials; germline mutation spectrum
BRCA2 (R3052W)	19563646	TRUE	TRUE	TRUE	p.R3052W in Abstract, Results and Discussion; pathogenicity assessment, classification model
BRCA2 (R3052W)	20513136	TRUE	TRUE	TRUE	p.Arg3052Trp in Abstract, Methods and Results; functional assay (human cell lines) and pathogenicity assessment
BRCA2 (R3052W)	29907814	TRUE	FALSE	TRUE	c.9154C>T in Results table; germline mutation testing, spectrum
BRCA2 (R3052W)	33078592	TRUE	FALSE	TRUE	p.Arg3052Trp in Results and Discussion; germline and tumor mutation testing and pathogenicity assessment
BRCA2 (R3052W)	29339979	TRUE	TRUE	TRUE	p.Arg3052Trp in Results table; germline mutation testing, frequency spectrum
BRCA2 (R2842L)	21990134	FALSE	TRUE	TRUE	R2842H in Main body table (Review type publication); pathogenicity assessment, classification model
BRCA2 (R2842L)	23108138	TRUE	TRUE	TRUE	R2842L in Results table and Discussion; germline mutation testing, functional assay (hamster cell line), pathogenicity assessment
BRCA2 (R2842L)	27907908	FALSE	TRUE	FALSE	Wrong variant: R2842H instead of R2842L
BRCA2 (R2842L)	24323938	TRUE	TRUE	TRUE	p.Arg2842Leu in Main body table; functional assay (human and non-human cell lines) and pathogenicity assessment
BRCA2 (R2842L)	29988080	TRUE	FALSE	TRUE	p.Arg2842Leu in Materials and methods table and Results; functional assay (mESC), pathogenicity assessment
BRCA2 (R2842L)	19043619	FALSE	TRUE	FALSE	Wrong variant: R2842H instead of R2842L
BRCA1 (D67E)	30696104	TRUE	FALSE	TRUE	p.Asp67Glu in Discussion; functional assay (E. Coli)
BRCA1 (D67E)	24507701	FALSE	TRUE	FALSE	Mentioned in the reference
BRCA1 (D67E)	30214240	FALSE	TRUE	FALSE	Mentioned in the reference
BRCA1 (D67E)	19865540	TRUE	FALSE	TRUE	Asp67Glu in Title, Abstract, Introduction, Results and Discussionand Experimental Section; functional assay (human cell line)
BRCA1 (D67E)	12203997	TRUE	FALSE	TRUE	Asp67Glu in Abstract; germline mutation testing, sample frequency spectrum
BRCA1 (D67E)	20967475	TRUE	TRUE	TRUE	D67E in Abstract; functional assay
BRCA1 (D67E)	22084573	TRUE	TRUE	TRUE	D67E in Abstract, Materials and methods, Results and Discussion; functional assay (E. Coli)
BRCA1 (A1830T)	21447777	TRUE	TRUE	TRUE	A1830T in Materials and Methods table; functional assay (yeast and mammalian cell lines) and pathegenicity assessment
BRCA1 (A1830T)	20516115	TRUE	TRUE	TRUE	A1830T in Results table; functional assay, pathogenicity assessment
BRCA1 (A1830T)	16354302	FALSE	TRUE	FALSE	No found occurence
BRCA2 (Q3066E)	33293522	TRUE	FALSE	TRUE	Q3066E in Results table; functional assay (mESC), pathogenicity assesement

Manual evaluation of the 50 documents retrieved only by Variomes

variant	PMID/PMCID	retrieved by Variomes	retrieved by LitVar	relevant	comment
BRCA1 (K45T)	24489791	TRUE	FALSE	TRUE	p.Lys45Thr in Discussion table; germline mutation testing, functional assay (human cell lines), pathogenicity assessment
BRCA2 (R174C)	22962691	TRUE	FALSE	TRUE	c.520C>T in Abstract; functional assay (patient-derived RNA)
BRCA2 (T2515I)	29988080	TRUE	FALSE	TRUE	p.Thr2515Ile in Materials and Methods table; functional assay (mouse embryonic stem cell - mESC), pathogenicity assessment
BRCA1 (M1775R)	26429972	TRUE	FALSE	TRUE	BRCA1M1775R in Results; functional assay (human cell lines)

BRCA2 (N372H)	29156805	TRUE	FALSE	TRUE	32906729 A C in Results table; circulating tumor mutation testing evaluation
BRCA2 (A2951T)	27223485	TRUE	FALSE	TRUE	p.A2951T in Results table; germline mutation testing, spectrum
BRCA2 (D1420N)	29458332	TRUE	FALSE	FALSE	Wrong variant: rs28897727 D1420Y instead of D1420N
BRCA2 (I2490T)	33293522	TRUE	FALSE	TRUE	I2490T in Results table; functional assay (mESC), pathogenicity assessment
BRCA1 (S1715R)	32322110	TRUE	FALSE	TRUE	S1715R in Appendix table; pathogenicity assessment, classification model and workflow
BRCA1 (C39Y)	33167967	TRUE	FALSE	FALSE	Wrong gene: VEGF −116G/A
BRCA1 (C61G)	17319787	TRUE	FALSE	TRUE	c.181T>G in Abstract; germline mutation testing, spectrum
BRCA1 (H239R)	27478808	TRUE	FALSE	FALSE	Wrong variant: rs80357396 A>T instead of A>G
BRCA1 (S1613C)	30541318	TRUE	FALSE	TRUE	rs1799966 in Abstract; meta-analysis, germline mutation testing, pathogenicity assessment
BRCA1 (S1613C)	21597964	TRUE	FALSE	TRUE	rs1799966, in Material and methods; germline mutation testing, haplotype structure
BRCA2 (S2988G)	30722038	TRUE	FALSE	TRUE	S2988G in Discussion; functional assay
BRCA2 (D1420N)	32039725	TRUE	FALSE	FALSE	Wrong variant: rs28897727 Asp1420Tyr instead of Asp1420Asn
BRCA1 (D1778G)	16026807	TRUE	FALSE	TRUE	D1778G in Abstract; germline mutation testing, spectrum
BRCA1 (L1407P)	32944641	TRUE	FALSE	TRUE	L1407P in Main body (Commentary type publication); functional assay (mouse)
BRCA1 (S1613C)	18830263	TRUE	FALSE	TRUE	rs1799966 in Main body table (Letter type publication); germline mutation testing, pathogenicity assessment
BRCA1 (T1685I)	29459887	TRUE	FALSE	TRUE	1685T → I in Main body table (Review type publication); functional assay and therapy response
BRCA2 (L2972W)	22771033	TRUE	FALSE	TRUE	L2972W in Results; functional assay (human and mouse cell lines)
BRCA1 (L1780P)	31788999	TRUE	FALSE	TRUE	Leu1780Pro in Results; germline mutation testing, sample type performance analysis
BRCA1 (D67Y)	32668560	TRUE	FALSE	FALSE	Wrong gene: RAD50 D67Y
BRCA1 (T1685I)	32322110	TRUE	FALSE	TRUE	T1685I in Appendix table; pathogenicity assessment, classification model and workflow
BRCA1 (P1238L)	21990134	TRUE	FALSE	TRUE	P1238L in Main body table (Review type publication); pathogenicity assessment, classification model
BRCA1 (D1739V)	31131967	TRUE	FALSE	TRUE	p.(Asp1739Val) in Methods table and Results and Discussion; pathogenicity assessment, classification model
BRCA1 (G1706A)	15689452	TRUE	FALSE	TRUE	G1706A in Abstract, Introduction, Methods, Results and Discussion; functional assay, germline mutation testing, pathogenicity assessment
BRCA1 (S1512I)	11836613	TRUE	FALSE	TRUE	S1512I in Abstract; germline mutation testing, family study
BRCA2 (D1420N)	29453630	TRUE	FALSE	FALSE	Wrong variant: rs28897727 Asp1420Tyr instead of Asp1420Asn
BRCA2 (R2034C)	30305041	TRUE	FALSE	TRUE	R2034C in Results; human cancer cell line mutation and functional testing
BRCA1 (R1699Q)	29459887	TRUE	FALSE	TRUE	1699R → Q in Main body table (Review type publication); functional assay and therapy response
BRCA1 (E1250K)	32272925	TRUE	FALSE	FALSE	Wrong variant: rs28897686 Glu1250* instead of Glu1250Lys
BRCA1 (R1699W)	33067490	TRUE	FALSE	TRUE	p.Arg1720Trp in Results table; germline mutation testing, spectrum
BRCA1 (R1726G)	21447777	TRUE	FALSE	TRUE	R1726G in Materials and methods table; functional assay (yeast and mammalian cell lines) and pathogenicity assessment
BRCA1 (C61G)	32463390	TRUE	FALSE	TRUE	c.181T>G in Results table and Discussion; germline mutation testing, occult cancer analysis
BRCA1 (R1495M)	23884293	TRUE	FALSE	FALSE	Wrong gene: AGL 484 G > T
BRCA2 (S1882*)	32806537	TRUE	FALSE	TRUE	Ser1882Ter in Results table; germline mutation testing, spectrum
BRCA2 (W2626C)	21520273	TRUE	FALSE	TRUE	W2626C in Abstract and Results; germline mutation testing, pathogenicity assessment
BRCA1 (R841W)	17233897	TRUE	FALSE	TRUE	Arg841Trp in Background; germline mutation testing, spectrum
BRCA1 (P1238R)	23313170	TRUE	FALSE	TRUE	rs28897688 in Results; germline mutation testing, radiation sensitivity
BRCA1 (G1738E)	31144781	TRUE	FALSE	TRUE	p.G1738E in Results table; pathogenicity assessment, classification model
BRCA1 (R1699Q)	29907814	TRUE	FALSE	TRUE	c.5096G>A in Results table; germline mutation testing, spectrum
BRCA2 (Q2384K)	21990134	TRUE	FALSE	TRUE	Q2384K in Main body table (Review type publication); pathogenicity assessment, classification model

BRCA2 (N372H)	32356124	TRUE	FALSE	TRUE	N372H in Discussion; germline mutation testing, family study
BRCA1 (R71G)	21520156	TRUE	FALSE	TRUE	c.211A>G in Abstract; germline mutation testing, pathogenicity assessment
BRCA1 (Q563*)	32164353	TRUE	FALSE	TRUE	c.1687C>T in Materials and Methods; germline mutation testing, family study
BRCA1 (S1040N)	22753008	TRUE	FALSE	TRUE	Ser1040Asn in Discussion table; functional assay (yeast and mouse cell lines), pathogenicity assessment
BRCA1 (R504H)	PMC3300770	TRUE	FALSE	TRUE	R504H in Results and Discussion; protein domain characterisation (E. Coli), pathogenicity assessment
BRCA1 (S1613C)	29657992	TRUE	FALSE	TRUE	rs1799966 in Material and methods table; germline mutation testing, treatment side effect prediction
BRCA2 (A2643G)	29988080	TRUE	FALSE	TRUE	p.Ala2643Gly in Materials and methods table; functional assay (mESC), pathogenicity assessment

$$publication\_score = score_{ElasticSearch} + 0.65 * (0.95 * score_{disease/gene} + 0.07 * score_{disease/variant} + 0.05 * score_{gene/variant}) + 0.1 * (0.97 * score_{density\text{-}disease} + 0.51 * score_{density\text{-}gene}\ 0.57 * score_{density\text{-}drug}) + 0.05 * (0.7 * score_{age} + 0.5 * score_{gender}) + 0.1 * (score_{keywords})$$

Equation used to score a document
